# Perspectives on therapeutic neutralizing antibodies against the Novel Coronavirus SARS-CoV-2

**DOI:** 10.7150/ijbs.45123

**Published:** 2020-03-15

**Authors:** Guangyu Zhou, Qi Zhao

**Affiliations:** 1 Faculty of Health Sciences, University of Macau, Taipa, Macau SPR, China.; 2 Institute of Translational Medicine, University of Macau, Taipa, Macau SPR, China.

**Keywords:** Neutralizing antibody, SARS-CoV-2, COVID-19, severe acute respiratory syndrome

## Abstract

A newly identified novel coronavirus (SARS-CoV-2) is causing pneumonia-associated respiratory syndrome across the world. Epidemiology, genomics, and pathogenesis of the SARS-CoV-2 show high homology with that of SARS-CoV. Current efforts are focusing on development of specific antiviral drugs. Therapeutic neutralizing antibodies (NAbs) against SARS-CoV-2 will be greatly important therapeutic agents for the treatment of coronavirus disease 2019 (COVID-19). Herein, the host immune responses against SARS-CoV discussed in this review provide implications for developing NAbs and understanding clinical interventions against SARS-CoV-2. Further, we describe the benefits, challenges and considerations of NAbs against SARS-CoV-2. Although many challenges exist, NAbs still offer a therapeutic option to control the current pandemic and the possible re-emergence of the virus in the future, and their development therefore remains a high priority.

## Introduction

The occurrence of coronavirus disease 2019 (COVID-19) cases in Wuhan city, Hubei province of China firstly emerged in December 2019. A newly identified novel coronavirus (SARS-CoV-2, formerly known as 2019-nCoV) is causing pneumonia-associated respiratory syndrome 1. After analysis of genome sequences of SARS-CoV-2 samples obtained from different infected patients, SARS-CoV-2 shares high sequence identity with SARS-CoV 2. Compared to SARS-CoV, transmitted from human-to-human of SARS-CoV-2 seems to be greater. As of February 2020, at least 25 countries reported >70,000 cases of SARS-CoV-2 infection. Patients infected with SARS-CoV-2 show typical pneumonia and severe lung damage 3. COVID-19 can be diagnosed by either clinical CT radiography or a laboratory real time Reverse Transcription-Polymerase Chain Reaction (RT-PCR) 4. Unfortunately, there are no specific antiviral drugs or vaccines currently. Several approaches can be suggested to control infections of SARS-CoV-2, including vaccines, monoclonal antibodies, oligonucleotides, peptides, interferon and small-molecule drugs 5. The antibody-mediated humoral response is crucial for preventing viral infections. A subset of these antibodies, which reduce viral infectivity by binding to the surface epitopes of viral particles and thereby blocking the entry of the virus to an infected cell, are defined as neutralizing antibodies (NAbs) 6. NAbs elicit their protective activities in three main steps. NAbs may prevent the attachment of the virion to its receptors on targeted cells, causing aggregation of virus particles. Further, the viruses are lysed through the constant (C) region of the antibody-mediated opsonization or complement activation 7. This review focuses on understanding immunopathogenesis of SARS-CoV-2 and addressing the benefits, challenges and considerations of neutralizing antibodies (NAbs).

## Similarity of SARS-CoV-2 and SARS-CoV in antigen and receptor recognition by host

**Figure 1:**
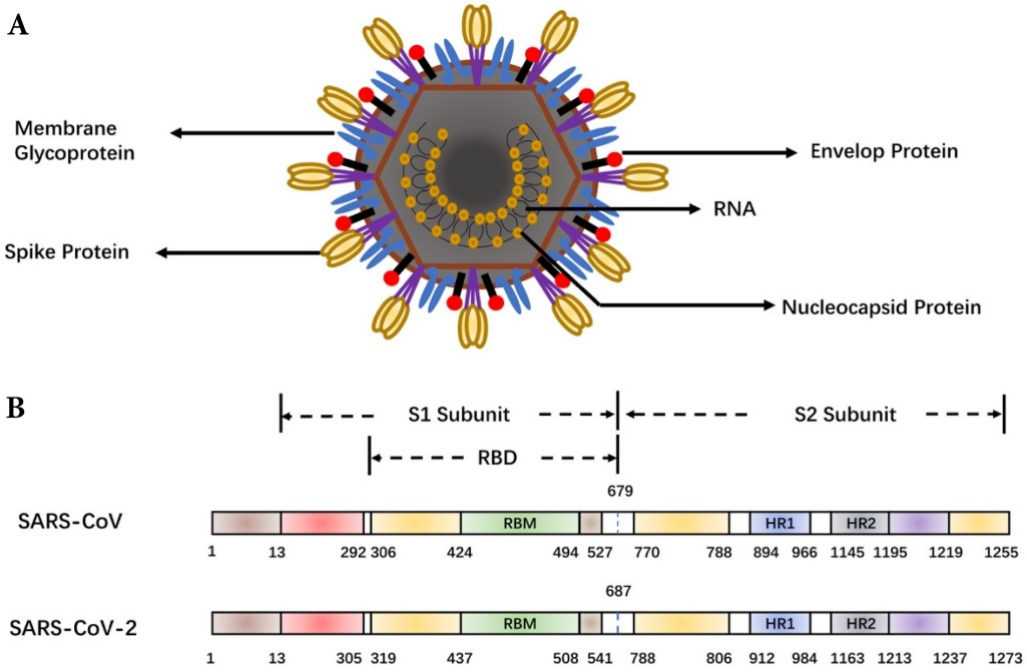
Schematic representation of the coronavirus and spike protein. **(A)** The coronavirus structure. The viral surface proteins (spike, envelope and membrane glycoproteins) are embedded in a lipid bilayer envelope. **(B)** Comparison of the spike (S) proteins of SARS-CoV and SARS-CoV-2. RBD, receptor-binding domain; RBM, receptor-binding motif; HR1/2, heptad repeat 1/2.

As shown in **Figure 1**, major structural proteins of SARS-CoV-2 include the spike (S), membrane (M) and envelop (E) and nucleic capsid (N) proteins 8. A coronavirus initiates cell fusion via attachment of the S protein with the receptor on the host cell surface. The viral nucleocapsid is delivered inside for subsequent replication. The S protein comprises two units, S1 and S2. The receptor-binding domain (RBD) within S1 directly interacts with host receptors 9. Structural and functional analysis of the SARS-CoV-2 shows that the SARS-CoV-2 S protein binds the Angiotensin-converting enzyme 2 (ACE2) receptor on human alveolar epithelial cells 10-12, suggesting SARS-CoV-2 uses the same receptor, ACE2, as SARS-CoV. However, the SARS-CoV-2 S protein binds ACE2 with higher affinity than SARS-CoV S 13. The high affinity of the S protein for human ACE2 may lead to the great human-to-human transmission of SARS-CoV-2. Due to the key role of the S protein, it is the main target for antibody-mediated neutralization.

## Innate and adaptive responses of human to SARS-CoV-2 and SARS-CoV

The clinical spectrum of the outcome of COVID-19 is highly variable, from mild flu-like symptoms to severe pneumonia. It is critical to take insights into cellular and humoral responses in SARS-CoV-2-induced COVID-19 14. Elucidation of SARS-CoV-2 immunopathogenesis is useful for developing passive antibody therapy, designing vaccines, and understanding of clinical drug interventions. However, the systemic landscape of the immune responses in patients with COVID-19 is unclear. Because the clinical features and immunopathogenesis of SARS-CoV-2 pose similarities with SARS-CoV 15, knowledge learned from SARS-CoV has important implications for understanding this new coronavirus.

Resistance to SARS-CoV infections is associated with both innate and adaptive immune responses 16. The innate immune response to SARS-CoV has not been completely defined 17. Some studies demonstrated that both macrophage and dendritic cell (DC) play the important roles for viral destruction and immune response induction in mucosal-associated lymphoid tissues 18. Due to homeostasis, macrophage and DC as vehicles seemed to disseminate viruses through the efferent lymphatic system. Meanwhile, activation of DC and macrophage by SARS-CoV led to excessive pro-inflammatory cytokine responses 19. A drastic elevation of inflammatory cytokines and chemokines was observed in the tissues and serum of SARS-CoV patients 20. The levels of, IFN-γ, IL-1β IL-6, IL-12, IL-8, MCP-1 and IP-10 are generally enhanced in the early infection and subsequently reduced in the recovery stage. Uncontrolled systemic inflammation (known as cytokine storm) further resulted in illness severity. The similar symptom of cytokine storm was observed in SARS-CoV-2 infection. The inflammatory cytokines and chemokines (IL-1β, IFN-γ, IP-10, and MCP-1), which may lead to activated T-helper-1 (Th1) cell responses, were upregulated 21,22. However, SARS-CoV-2 patients secreted excessive IL-4 and IL-10 that may suppress inflammation via T-helper-2 (Th2) 14. It differs from SARS-CoV infection. Further studies are necessary to elucidate innate responses in pathogenesis of SARS-CoV-2.

The adaptive immune response mainly consists of cellular (T cell) and humoral (B cell) responses. T cell-mediated responses in SARS-CoV infection have been well elucidated 23. Both CD4+ and CD8+ T-cells provided broad and long-term protection. CD4+ T cells promoted the proliferation of neutralizing antibodies, whereas CD8+ T cells were responsible for the destruction of viral infected cells. Although all SARS‐CoV surface proteins, including S, M, E, and N proteins were involved in T cell responses, S protein contributed to the most T-cell recognition epitopes. Overall frequency of CD8+T cell response predominates over CD4+T cell response. Lymphopenia occurred in both SARS-CoV and SARS-CoV-2 infections 14,24,25. The reduction of CD4+ and CD8+ T cells is commonly associated with lymphopenia. It will be interesting to elucidate T-cell mediated response in SARS-CoV-2 infection that may provide important hints for the design of the vaccine composed of viral structural proteins. On the other hand, patients with SARS-CoV infection had the strong humoral immune response to SARS-CoV 26,27. Serum IgG, IgM, and IgA responses to SARS-CoV appeared in patients after primary SARS infection 28. Neutralizing IgGs played a major role in the neutralization of the SARS-CoV. IgGs reached the peak in serum during the convalescent phase and diminished after recovery 29. Memory B cells still provided the long-term protection in associated with cellular immune responses 30. Despite markedly reducing virus replication, anti-S protein neutralizing IgGs could be associated with fatal acute lung injury through promoting IL-8/MCP-1 production and inflammatory macrophage accumulation 31. These studies may provide important implications for observing IgG response in patients with SARS-CoV-2.

## Advances in the development of neutralizing antibodies to SARS-CoV

NAbs provide important specific immune defense against viral infections in patients 32, 33,34. Numbers of antiviral NAbs have been developed in recent years, and some are now in clinical development. The role and importance of NAbs in protection from SARS-CoV infection has been thoroughly reviewed elsewhere 7,35-38. Entry of SARS-CoV into the host cell is mediated by the attachment of S protein and ACE-2 receptor. The S protein is the major inducer of NAbs. Particularly, RBD within S1 unit is the most critical target for SARS-CoV NAbs 39. Such NAbs can interrupt the interaction of RBD and its receptor ACE2. Most of NAbs have been identified to recognize RBD region 40-46. Interestingly, some NAbs still showed to recognize epitopes on S2 unit 47, suggesting that other mechanisms could be involved in the neutralization. At last virus clearance was mediated by antibody-dependent opsonization or complement activation 7. These NAbs against SARS-CoV are summarized in **Table 1**.

Phage display has been used to identify neutralizing human monoclonal antibodies against SARS-CoV from both naïve and immune antibody libraries. The selected antibodies, 80R 40, CR3014 41, CR3022 42, m396 43, blocked the binding of S1 domain and ACE2. 80R, CR3013 and m396 showed virus neutralization and prophylaxis capability in either vitro or animal models. Although CR3022 did not showed much neutralization alone, the mixture of CR3022 and CR3014 showed neutralization of SARS-CoV in a synergistic effect due to recognition of different epitopes on RBD 42. A method for Epstein-Barr virus (EBV) transformation of human B cells was used to isolate NAbs. Six groups of NAbs, which were divided based on differential neutralization of SARS-CoV variants, have been successfully identified from memory B cells from SARS-CoV infected patients 30. Furthermore, transgenic mice with human immunoglobulin genes have been used to produce NAbs against SARS-CoV by antigen immunization. Two NAbs, 201 and 68, were identified from transgenic mice 44,45. They were effective for virus prophylaxis in animal models. On the other hand, several NAbs, B1 46, 1F8 and 5E9 47, against epitopes on SARS-CoV S2 still showed effectiveness in neutralization.

## Perspectives on the development of neutralizing antibodies against SARS-CoV-2

The simplest and most direct approach to combating SARS-CoV-2 during the outbreak would be to use plasma from the convalescent patients 48. Polyclonal NAbs could be induced in some convalescent patients and will be effective in treating SARS-CoV-2 12. These NAbs can provide passive immune responses to viral infection. Indeed, both SARS and Ebola patients received the treatment of convalescent plasma 49,50. However, the outcomes of passive plasma therapy are unpredictable due to variability of sera in different patients.

**Figure 2:**
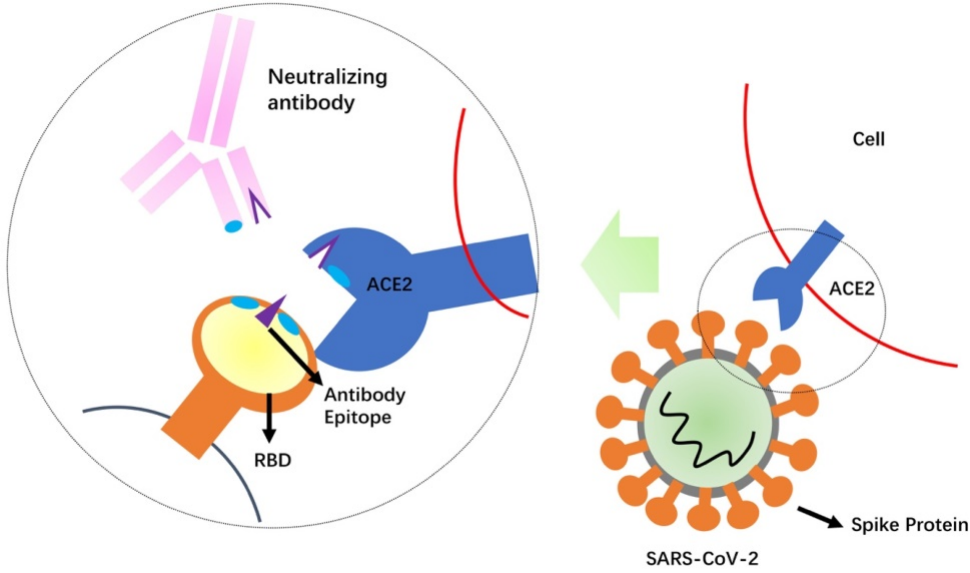
Schematic mechanism of the neutralizing antibodies. Competition of the neutralizing antibody with the receptor (ACE2) for binding to the receptor-binding domain (RBD) of the SARS-CoV-2 Spike protein is shown. The protruding portion (violet) of RBD is both the ACE2 receptor-binding site and the antibody epitope.

Development of NAbs against SARS-CoV-2 is a relatively rapid approach to obtain the standardized agents that control re-emergence of COVID-19 51. The SARS-CoV-2 S protein is likely important target for developing NAbs to block binding and fusion of SARS-CoV-2 (**Figure 2**). SARS-CoV-2 seems to use the same cell entry receptor, ACE2, as the SARS-CoV because ACE2 shows binding to RBD of both SARS-CoV and SARS-CoV-2 11. However, a recent study demonstrates that SARS-CoV-2 S protein binds ACE2 with higher affinity than SARS-CoV (10- to 20-folder) 13, suggesting its recognition to ACE2 could be different with SARS-CoV. Although SARS-CoV-2 shows the high homology with SARS-CoV, antibody cross-reactivity is limited between the two virus S proteins. Several published SARS-CoV NAbs do not have appreciable binding to SARS-CoV-2 S protein 13,52. A recent study shows that a SARS-CoV antibody, CR3022, binds to SARS-CoV-2 RBD 52, but its neutralization capability is uncertain. Cocktail of NAbs has showed the stronger neutralization than alone in treatment of both Ebola and SARS viruses 47,53. This finding suggests that a cocktail antibody approach for SARS-CoV-2 could be undertaken. Therefore, it will be very meaningful to generate NAbs targeting different epitopes on SARS-CoV-2. Combination of several potent NAbs could decrease the probability for escape virus isolates with decreased sensitivity to neutralization.

Computational simulation of antibody-antigen complexes has been used to guide the design of therapeutic antibodies 54-56. Numbers of antibody structures (currently around 2,000 depositions) are available in the Protein Data Bank [PDB]. Based on these PDB data, the comparative model of an antibody onto the viral surface antigen can be predicted. The key residues between RBD and NAbs can be identified to provide important implications for the vaccines against SARS-CoV-2. The key residues of interface between an antibody and the antigen can be optimized to produce high affinity 57. Several recent computer docking models have been used to predict the interaction between S protein and human ACE2 10 or antibodies 52. The studies revealed the important discovery that SARS-CoV-specific CR3022 antibody could cross-react to SARS-CoV-2.

## Conclusion

The availability of therapeutic NAbs against SARS-CoV-2 will offer benefits for the control of the current pandemic and the possible re-emergence of the virus in the future, and their development therefore remains a high priority. The efforts of NAb development will surely be an area of intense research in the coming months and even years. Currently, several strategies are used in the clinic or under development, such as viral-targeting therapeutics and host-targeting agents (such as interferons, glucocorticoids) for the treatment of COVID-19. As compared with these therapeutic strategies, NAbs appear to be more specific for virions. Understanding of action mechanisms of NAbs may provide valuable implications for the rapid development of antibody therapy and vaccine for SARS-CoV-2. However, the development of NAb-based therapeutics is a time-consuming and laborious process. To date, no NAb agents for either SARS-CoV or (Middle East Respiratory Syndrome Coronavirus) MERS-CoV are available in the market. Meanwhile, a note of caution is that the effect of antibody immune response in protecting against pulmonary pathogenesis of SARS-CoV is controversial 31. Some patients who died of SARS showed the strong NAb responses and pulmonary proinflammatory accumulation, suggesting NAbs could be associated with fatal acute lung injury. Therefore, it is important to take insight into humoral and cellular responses of SARS-CoV-2 when antiviral immunotherapy is developed.

## Figures and Tables

**Table 1:** Neutralizing antibodies against SARS-CoV

Neutralizing antibody	Identification Method	Target Region	Animal model	Reference
80R	Phage display	S1 domain 426-492	Mouse	40
CR3014	Phage display	S1 domain 318-510	Ferret	41
CR3022	Phage display	S1 domain 318-510	NA	42
m396	Phage display	S protein	Mouse	43
B1	Phage display	S2 domain 1023-1189	NA	46
Group I (S132, S228.11)	EBV transformed B cells	N-terminal RBD	NA	30
Group II (S111.7, S224.17)	EBV transformed B cells	S1 domain 318-510	NA	30
Group III (S3.1, S127.6, S217.4, S222.1, S237.1)	EBV transformed B cells	S1 domain 318-510	Mouse (S.1)	30
Group IV (S110.4, S218.9, S223.4, S225.12, S226.10, S231.19, S232.17, S234.6)	EBV transformed B cells	S1 domain 318-510	NA	30
Group V (S124.5, S219.2)	EBV transformed B cells	ND	NA	30
Group VI (S109.8, S215.17, S227.14, S230.15)	EBV transformed B cells	S1 domain 318-510	Mouse	30
201	HuMAb-Mouse^®^	S1 domain 490-510	Mouse	44, 45
68	HuMAb-Mouse^®^	S1 domain 130-150	Mouse	44, 45
1F8	XenoMouse®	S2 domain HR1	NA	47
5E9	XenoMouse®	S2 domain HR2	NA	47

## References

[1] Zhu N, Zhang D, Wang W, Li X, Yang B, Song J (2020). A Novel Coronavirus from Patients with Pneumonia in China, 2019. N Engl J Med.

[2] Lu R, Zhao X, Li J, Niu P, Yang B, Wu H (2020). Genomic characterisation and epidemiology of 2019 novel coronavirus: implications for virus origins and receptor binding. Lancet.

[3] Jiang S, Xia S, Ying T, Lu L (2020). A novel coronavirus (2019-nCoV) causing pneumonia-associated respiratory syndrome. Cell Mol Immunol.

[4] Xie X, Zhong Z, Zhao W, Zheng C, Wang F, Liu J (2020). Chest CT for Typical 2019-nCoV Pneumonia: Relationship to Negative RT-PCR Testing. Radiology.

[5] Li G, Clercq E (2020). Therapeutic options for the 2019 novel coronavirus (2019-nCoV). Nature Reviews Drug Discovery.

[6] Klasse PJ (2014). Neutralization of Virus Infectivity by Antibodies: Old Problems in New Perspectives. Adv Biol.

[7] Coughlin MM, Prabhakar BS (2012). Neutralizing human monoclonal antibodies to severe acute respiratory syndrome coronavirus: target, mechanism of action, and therapeutic potential. Rev Med Virol.

[8] Schoeman D, Fielding BC (2019). Coronavirus envelope protein: current knowledge. Virol J.

[9] Li F (2012). Evidence for a common evolutionary origin of coronavirus spike protein receptor-binding subunits. J Virol.

[10] Xu X, Chen P, Wang J, Feng J, Zhou H, Li X (2020). Evolution of the novel coronavirus from the ongoing Wuhan outbreak and modeling of its spike protein for risk of human transmission. Sci China Life Sci.

[11] Wan Y, Shang J, Graham R, Baric RS, Li F (2020). Receptor recognition by novel coronavirus from Wuhan: An analysis based on decade-long structural studies of SARS. J Virol.

[12] Zhou P, Yang XL, Wang XG, Hu B, Zhang L, Zhang W (2020). A pneumonia outbreak associated with a new coronavirus of probable bat origin. Nature.

[13] Wrapp D, Wang N, Corbett KS, Goldsmith JA, Hsieh CL, Abiona O (2020). Cryo-EM structure of the 2019-nCoV spike in the prefusion conformation. Science.

[14] Huang C, Wang Y, Li X, Ren L, Zhao J, Hu Y (2020). Clinical features of patients infected with 2019 novel coronavirus in Wuhan, China. Lancet.

[15] Liu J, Zheng X, Tong Q, Li W, Wang B, Sutter K (2020). Overlapping and discrete aspects of the pathology and pathogenesis of the emerging human pathogenic coronaviruses SARS-CoV, MERS-CoV, and 2019-nCoV. J Med Virol.

[16] Li C, Xu X (2010). Host Immune Responses to SARS Coronavirus in Humans. Molecular Biology of the SARS-Coronavirus. Heidelberg: Springer.

[17] Frieman M, Heise M, Baric R (2008). SARS coronavirus and innate immunity. Virus Res.

[18] Liu L, Wei Q, Nishiura K, Peng J, Wang H, Midkiff C (2016). Spatiotemporal interplay of severe acute respiratory syndrome coronavirus and respiratory mucosal cells drives viral dissemination in rhesus macaques. Mucosal Immunol.

[19] Tseng CT, Perrone LA, Zhu H, Makino S, Peters CJ (2005). Severe acute respiratory syndrome and the innate immune responses: modulation of effector cell function without productive infection. J Immunol.

[20] Thiel V, Weber F (2008). Interferon and cytokine responses to SARS-coronavirus infection. Cytokine Growth Factor Rev.

[21] Wang D, Hu B, Hu C, Zhu F, Liu X, Zhang J (2020). Clinical Characteristics of 138 Hospitalized Patients With 2019 Novel Coronavirus-Infected Pneumonia in Wuhan, China. JAMA. 2020; doi: 10.1001/jama.

[22] Chen L, Liu HG, Liu W, Liu J, Liu K, Shang J (2020). [Analysis of clinical features of 29 patients with 2019 novel coronavirus pneumonia]. Zhonghua Jie He He Hu Xi Za Zhi.

[23] Janice Oh HL, Ken-En Gan S, Bertoletti A, Tan YJ (2012). Understanding the T cell immune response in SARS coronavirus infection. Emerg Microbes Infect.

[24] Panesar NS (2003). Lymphopenia in SARS. Lancet.

[25] Hui DS, E IA, Madani TA, Ntoumi F, Kock R, Dar O (2020). The continuing 2019-nCoV epidemic threat of novel coronaviruses to global health - The latest 2019 novel coronavirus outbreak in Wuhan, China. Int J Infect Dis.

[26] Li G, Chen X, Xu A (2003). Profile of specific antibodies to the SARS-associated coronavirus. N Engl J Med.

[27] Cheng M, Chan CW, Cheung RC, Bikkavilli RK, Zhao Q, Au SW (2005). Cross-reactivity of antibody against SARS-coronavirus nucleocapsid protein with IL-11. Biochem Biophys Res Commun.

[28] Woo PC, Lau SK, Wong BH, Chan KH, Chu CM, Tsoi HW (2004). Longitudinal profile of immunoglobulin G (IgG), IgM, and IgA antibodies against the severe acute respiratory syndrome (SARS) coronavirus nucleocapsid protein in patients with pneumonia due to the SARS coronavirus. Clin Diagn Lab Immunol.

[29] Cao WC, Liu W, Zhang PH, Zhang F, Richardus JH (2007). Disappearance of antibodies to SARS-associated coronavirus after recovery. N Engl J Med.

[30] Traggiai E, Becker S, Subbarao K, Kolesnikova L, Uematsu Y, Gismondo MR (2004). An efficient method to make human monoclonal antibodies from memory B cells: potent neutralization of SARS coronavirus. Nat Med.

[31] Liu L, Wei Q, Lin Q, Fang J, Wang H, Kwok H (2019). Anti-spike IgG causes severe acute lung injury by skewing macrophage responses during acute SARS-CoV infection. JCI Insight.

[32] Nie Y, Wang G, Shi X, Zhang H, Qiu Y, He Z (2004). Neutralizing antibodies in patients with severe acute respiratory syndrome-associated coronavirus infection. J Infect Dis.

[33] Wang Y, Shan Y, Gao X, Gong R, Zheng J, Zhang XD (2018). Screening and expressing HIV-1 specific antibody fragments in Saccharomyces cerevisiae. Mol Immunol.

[34] Li D, Liu J, Zhang L, Xu T, Chen J, Wang L (2015). N-terminal residues of an HIV-1 gp41 membrane-proximal external region antigen influence broadly neutralizing 2F5-like antibodies. Virol Sin.

[35] Zhang MY, Choudhry V, Xiao X, Dimitrov DS (2005). Human monoclonal antibodies to the S glycoprotein and related proteins as potential therapeutics for SARS. Curr Opin Mol Ther.

[36] Prabakaran P, Zhu Z, Xiao X, Biragyn A, Dimitrov AS, Broder CC (2009). Potent human monoclonal antibodies against SARS CoV, Nipah and Hendra viruses. Expert Opin Biol Ther.

[37] Zhu Z, Prabakaran P, Chen W, Broder CC, Gong R, Dimitrov DS (2013). Human monoclonal antibodies as candidate therapeutics against emerging viruses and HIV-1. Virol Sin.

[38] Jin Y, Lei C, Hu D, Dimitrov DS, Ying T (2017). Human monoclonal antibodies as candidate therapeutics against emerging viruses. Front Med.

[39] Wong SK, Li W, Moore MJ, Choe H, Farzan M (2004). A 193-amino acid fragment of the SARS coronavirus S protein efficiently binds angiotensin-converting enzyme 2. J Biol Chem.

[40] Sui J, Li W, Murakami A, Tamin A, Matthews LJ, Wong SK (2004). Potent neutralization of severe acute respiratory syndrome (SARS) coronavirus by a human mAb to S1 protein that blocks receptor association. Proc Natl Acad Sci U S A.

[41] van den Brink EN, Ter Meulen J, Cox F, Jongeneelen MA, Thijsse A, Throsby M (2005). Molecular and biological characterization of human monoclonal antibodies binding to the spike and nucleocapsid proteins of severe acute respiratory syndrome coronavirus. J Virol.

[42] ter Meulen J, van den Brink EN, Poon LL, Marissen WE, Leung CS, Cox F (2006). Human monoclonal antibody combination against SARS coronavirus: synergy and coverage of escape mutants. PLoS Med.

[43] Zhu Z, Chakraborti S, He Y, Roberts A, Sheahan T, Xiao X (2007). Potent cross-reactive neutralization of SARS coronavirus isolates by human monoclonal antibodies. Proc Natl Acad Sci U S A.

[44] Coughlin M, Lou G, Martinez O, Masterman SK, Olsen OA, Moksa AA (2007). Generation and characterization of human monoclonal neutralizing antibodies with distinct binding and sequence features against SARS coronavirus using XenoMouse. Virology.

[45] Greenough TC, Babcock GJ, Roberts A, Hernandez HJ, Thomas WD Jr, Coccia JA (2005). Development and characterization of a severe acute respiratory syndrome-associated coronavirus-neutralizing human monoclonal antibody that provides effective immunoprophylaxis in mice. J Infect Dis.

[46] Duan J, Yan X, Guo X, Cao W, Han W, Qi C (2005). A human SARS-CoV neutralizing antibody against epitope on S2 protein. Biochem Biophys Res Commun.

[47] Elshabrawy HA, Coughlin MM, Baker SC, Prabhakar BS (2012). Human monoclonal antibodies against highly conserved HR1 and HR2 domains of the SARS-CoV spike protein are more broadly neutralizing. PLoS One.

[48] Kruse RL (2020). Therapeutic strategies in an outbreak scenario to treat the novel coronavirus originating in Wuhan, China. F1000Research.

[49] Cheng Y, Wong R, Soo YO, Wong WS, Lee CK, Ng MH (2005). Use of convalescent plasma therapy in SARS patients in Hong Kong. Eur J Clin Microbiol Infect Dis.

[50] Kraft CS, Hewlett AL, Koepsell S, Winkler AM, Kratochvil CJ, Larson L (2015). The Use of TKM-100802 and Convalescent Plasma in 2 Patients With Ebola Virus Disease in the United States. Clin Infect Dis.

[51] Jiang S, Du L, Shi Z (2020). An emerging coronavirus causing pneumonia outbreak in Wuhan, China: calling for developing therapeutic and prophylactic strategies. Emerg Microbes Infect.

[52] Tian X, Li C, Huang A, Xia S, Lu S, Shi Z (2020). Potent binding of 2019 novel coronavirus spike protein by a SARS coronavirus-specific human monoclonal antibody. Emerg Microbes Infect.

[53] Group PIW, Multi-National PIIST, Davey RT Jr, Dodd L, Proschan MA, Neaton J (2016). A Randomized, Controlled Trial of ZMapp for Ebola Virus Infection. N Engl J Med.

[54] Zhao Q, Ahmed M, Tassev DV, Hasan A, Kuo TY, Guo HF (2015). Affinity maturation of T-cell receptor-like antibodies for Wilms tumor 1 peptide greatly enhances therapeutic potential. Leukemia.

[55] Li D, Gong R, Zheng J, Chen X, Dimitrov DS, Zhao Q (2017). Engineered antibody CH2 domains binding to nucleolin: Isolation, characterization and improvement of aggregation. Biochem Biophys Res Commun.

[56] Zhao Q, Ahmed M, Guo HF, Cheung IY, Cheung NK (2015). Alteration of Electrostatic Surface Potential Enhances Affinity and Tumor Killing Properties of Anti-ganglioside GD2 Monoclonal Antibody hu3F8. J Biol Chem.

[57] Barderas R, Desmet J, Timmerman P, Meloen R, Casal JI (2008). Affinity maturation of antibodies assisted by in silico modeling. Proc Natl Acad Sci U S A.

